# Exploring the Cost Effectiveness of Shared Decision Making for Choosing between Disease-Modifying Drugs for Relapsing-Remitting Multiple Sclerosis in the Netherlands: A State Transition Model

**DOI:** 10.1177/0272989X20961091

**Published:** 2020-11-11

**Authors:** Ingrid E. H. Kremer, Mickael Hiligsmann, Josh Carlson, Marita Zimmermann, Peter J. Jongen, Silvia M. A. A. Evers, Svenja Petersohn, Xavier G. L. V. Pouwels, Nick Bansback

**Affiliations:** Department of Health Services Research, CAPHRI Care and Public Health Research Institute, Maastricht University, Maastricht, Limburg, The Netherlands; School of Population and Public Health, University of British Columbia, Vancouver, BC, Canada; Department of Health Services Research, CAPHRI Care and Public Health Research Institute, Maastricht University, Maastricht, Limburg, The Netherlands; Department of Pharmacy, University of Washington, Seattle, WA, USA; Institute for Disease Modeling, Bellevue, WA, USA; MS4 Research Institute, Nijmegen, The Netherlands; Department of Community and Occupational Medicine, University Medical Center Groningen, Groningen, The Netherlands; Department of Health Services Research, CAPHRI Care and Public Health Research Institute, Maastricht University, Maastricht, Limburg, The Netherlands; Centre for Economic Evaluations, Trimbos Institute, Utrecht, The Netherlands; Department of Clinical Epidemiology and Medical Technology Assessment, Maastricht University Medical Centre, Maastricht, Limburg, The Netherlands; Care and Public Health Research Institute, Maastricht University, Maastricht, The Netherlands; Department of Health Technology & Services Research, Faculty of Behavioral, Management & Social Sciences, University of Twente, Enschede, The Netherlands; School of Population and Public Health, University of British Columbia, Vancouver, BC, Canada

**Keywords:** disease-modifying drugs, early economic evaluation, multiple sclerosis, shared decision making, state transition model

## Abstract

**Background:**

Up to 31% of patients with relapsing-remitting multiple sclerosis (RRMS) discontinue treatment with disease-modifying drug (DMD) within the first year, and of the patients who do continue, about 40% are nonadherent. Shared decision making may decrease nonadherence and discontinuation rates, but evidence in the context of RRMS is limited. Shared decision making may, however, come at additional costs. This study aimed to explore the potential cost-effectiveness of shared decision making for RRMS in comparison with usual care, from a (limited) societal perspective over a lifetime.

**Methods:**

An exploratory economic evaluation was conducted by adapting a previously developed state transition model that evaluates the cost-effectiveness of a range of DMDs for RRMS in comparison with the best supportive care. Three potential effects of shared decision making were explored: 1) a change in the initial DMD chosen, 2) a decrease in the patient’s discontinuation in using the DMD, and 3) an increase in adherence to the DMD. One-way and probabilistic sensitivity analyses of a scenario that combined the 3 effects were conducted.

**Results:**

Each effect separately and the 3 effects combined resulted in higher quality-adjusted life years (QALYs) and costs due to the increased utilization of DMD. A decrease in discontinuation of DMDs influenced the incremental cost-effectiveness ratio (ICER) most. The combined scenario resulted in an ICER of €17,875 per QALY gained. The ICER was sensitive to changes in several parameters.

**Conclusion:**

This study suggests that shared decision making for DMDs could potentially be cost-effective, especially if shared decision making would help to decrease treatment discontinuation. Our results, however, may depend on the assumed effects on treatment choice, persistence, and adherence, which are actually largely unknown.

Multiple sclerosis (MS) is a progressive demyelinating and degenerative disease of the central nervous system, causing physical and cognitive disabilities and a substantial loss of quality of life.^1–3^ In the Netherlands, 88 per 100,000 people are diagnosed with MS, and each year another 5 per 100,000 people will receive the diagnosis.^4^ Different courses of MS can be distinguished, the majority of patients (approximately 89%) having the relapsing-remitting course of MS (RRMS) at onset.^4^ RRMS is characterized by relapses (i.e., the occurrence of new symptoms or exacerbations of existing symptoms), followed by remissions, in which the symptoms recover completely or incompletely, and stable periods.^5,6^ RRMS patients could transition to a secondary progressive MS (SPMS) course, characterized by a transition from relapses and remissions to a gradual continuous worsening of symptoms.^5,6^ In the Netherlands, cost of MS summed up to €204.2 million (0.21% of total health care expenditures) in 2017, consisting of 46% of hospital and specialist care and 35% of drug treatment.^7^

Currently, a large number of disease-modifying drugs (DMDs) are available to reduce the number and severity of relapses in patients with RRMS.^8,9^ By reducing the number and severity of relapses, DMDs also delay the accumulation of disabilities. Patients with RRMS most often have several options in the treatment with DMDs, including the choice to refrain from DMD use. The use of DMDs can be burdensome for patients due to side effects and administration regimens; these characteristics differ between DMDs.

Decision making about treatment for RRMS is therefore difficult, and the patient should be involved^10^ for several reasons. First, more efficacious DMDs are associated with a higher risk of severe or life-threatening adverse events, resulting in these DMDs typically being indicated for patients with high disease activity.^11^ Moreover, patients experience the burden of administration and common side effects differently, which puts a different value on DMDs with certain attributes.^12^ Second, health care providers making a treatment decision without consulting the patient often make inaccurate assessments of the patient’s preferences for treatment options, instead basing assessment on personal preferences and experience.^13^ Third, persistence with and adherence to treatment regimens are suboptimal among many patients.^14–17^ Treatment persistence refers to “the length of time between initiation and the last dose, which immediately precedes discontinuation,” with discontinuation referring to the patients stopping the medication.^18^ With treatment adherence, we refer to the “implementation” component according to the taxonomy by Vrijens et al.^18^: “the extent to which a patient’s actual dosing corresponds to the prescribed dosing regimen, from initiation until the last dose.”^19^ Real-world studies in RRMS show that discontinuation rates in the first year after DMD treatment initiation range between 10% and 31%.^15–17^ Of the people who do persist, only 60% of patients taking injectable or orally administered DMDs were reported to have optimal adherence.^14^

Shared decision making is an approach that can help explicitly to integrate informed patient’s preferences for treatment options into clinical decisions.^20^ Health care professionals enable the patient to develop informed preferences for treatment options by sharing information. In turn, the patient shares his or her preferences with the health care provider. Consecutively, the patient and health care professional discuss the best matching treatment options, considering the patient’s preferences and the best available evidence, to make a treatment decision together.^20^ Shared decision making is often supported by patient decision aids, which inform patients about their options and help them to understand and express their preferences.^21^ Patient decision aids have been shown to facilitate shared decision making^21^ and could improve treatment adherence.^22^

The implementation of shared decision making with or without a patient decision aid can also have implications for resources. Trenaman et al.^23^ distinguish 3 categories for how resources could be affected through the use of a patient decision aid, potentially increasing or decreasing costs: 1) delivery of the patient decision aid and its effect on consultation time, 2) short-term costs because patients may opt more often for more or less expensive options, or 3) long-term costs because of a postponed intervention or changes in persistence and adherence. For example, implementation of shared decision making with or without patient decision aids could increase consultation time and thus increase costs.^21^ Furthermore, cost reductions in the short term have been reported for one-off interventions because patients chose less invasive and less costly interventions.^24,25^ In addition, patient education and value elicitation could support patients in developing a more persistent and adherent attitude toward taking a particular medication. As a result, health outcomes would improve and utilization of health care, and consequently costs, would decrease.^26^ The consequences for costs may depend, however, on the treatment decision and the context.^23^ While there is less evidence on the consequences of shared decision making and patient decision aids regarding persistence, adherence, health outcomes, and costs for chronic diseases requiring long-term treatment,^23^ various efforts are under way to create training and tools for MS.

An investment in shared decision making has an opportunity cost, diverting funds used for other aspects of the health care system, including the budgets used to pay for drugs. It is therefore important to understand the cost-effectiveness of shared decision making, similar to how decision makers assess the cost-effectiveness of drugs for reimbursement decisions.^27^ Accordingly, this study aimed to evaluate the potential lifetime cost-effectiveness of shared decision making with regard to DMDs for RRMS in comparison with usual care from a societal perspective. The study had an exploratory nature to determine the headroom for implementation of shared decision making for DMDs for RRMS. Therefore, we also estimated the maximum costs at which shared decision making remains cost-effective. Although evidence for the effects of shared decision making is limited, the results of this early economic evaluations could inform decision makers and clinicians about the potential value of implementing shared decision making in clinical practice before wide implementation of the approach is realized. By explicitly modeling the different consequences of shared decision making (i.e., the costs of delivery of shared decision making, treatment choice, persistence and adherence) on relapses, quality-adjusted life years (QALYs), and costs, this study also aimed to reveal which effects drive the cost-effectiveness. Results could underline the need to develop effective interventions for implementing shared decision making (e.g., patient decision aids) and to help in designing future trial-based and model-based economic evaluations of shared decision making and other interventions focused on improving persistence and adherence during long-term treatment.

## Methods

We adapted a state transition model developed by the Institute for Clinical and Economic Review, an independent and nonpartisan research institute, in the United States.^28^ The model evaluated the cost-effectiveness of a range of DMDs for MS.^28^ The model structure and inputs, based on various other models,^29–35^ have previously been validated through rounds of public comments, cross-validation with other models, and sensitivity analyses.^28^ We modified the model to assess the cost-effectiveness of implementing shared decision making regarding DMDs choice and estimated the potential societal costs, QALYs, and incremental cost-effectiveness using a Dutch perspective and following clinical practice and guidelines for economic evaluations in health care.^36^ For reporting, we followed the Consolidated Health Economic Evaluation Reporting Standards (CHEERS).^37^

### Population

The modeled population were adults with RRMS without prior experience with DMDs in the Netherlands. These patients had a mean age of RRMS onset of 37 years.^38,39^ Within this population, 29% were male.^38,39^

### Intervention and Comparator

Implementation of shared decision making in the intervention group was compared with usual care in the control group. In the intervention group, the health care professional applies the principles of shared decision making with the patient during the decision making process about DMDs. Patient decision aids are commonly used during or between 2 consultations to educate patients about their treatment options and to help clarify personal values regarding the treatment options.^21^ During a (follow-up) consultation, the patient and health care professional discuss the treatment options and the patient’s preferences to make an informed and shared decision on treatment.^20^ In the control group, we assume that usual care decisions are mostly made in accordance with the health care professional’s judgment of what fits best with the patient’s needs, with little attempt to determine or acknowledge what the patient’s preferences might be with regard to the decision. In both groups, patients would be prescribed a treatment with a DMD or no active treatment (best supportive care).

### Model Structure

**Figure 1:**
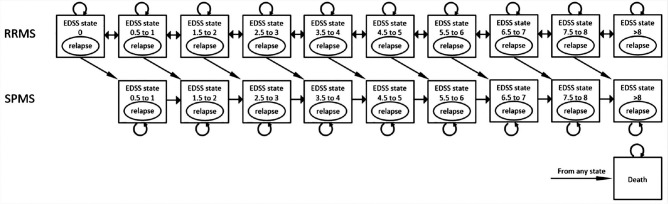
Health state transition model structure for multiple sclerosis course, adapted from Zimmermann et al.^40^ Patients with RRMS enter the model in any health states defined by neurologic impairment measured with the EDSS. Higher EDSS scores indicate worse neurologic impairment. During a cycle, patients can stay in their current health state or transition to a consecutive lower or higher health state. Patients can also transition to SPMS or die. In any health state, patients can experience a relapse. EDSS, Expanded Disability Status Scale; RRMS, relapsing-remitting multiple sclerosis; SPMS, secondary progressive multiple sclerosis.

To model the disease course of RRMS and the risk for progression to SPMS, 20 health states (i.e., 10 health states for RRMS and 9 health states for SPMS and death) (Figure 1), defined by the Expanded Disability Status Scale (EDSS), were included according to the model described by Zimmermann et al.^40^ The EDSS, administered by a physician, measures neurological impairment in patients with MS on an ordinal scale from 0 (no impairment) to 10 (death), defined by the degree of impairment in functional systems, such as pyramidal, cerebellar, sensory, and visual functions, and the degree of ambulatory problems.^41^ Patients with RRMS could enter the model in health states with an EDSS score between 0 and 9. During a cycle of 12 months, patients could improve or worsen in RRMS health states, remain stable, or progress to SPMS. The transition probabilities were estimated based on natural history.^33^ The effectiveness of each DMD is modeled through applying a relative risk for each DMD to the transition probabilities.^40^ In the SPMS health states, patients could progress to a higher EDSS state or remain in the same health state (i.e., patients could not improve). Patients could experience a relapse or die in each health state. Patients transitioning to SPMS were assumed to continue treatment based on current clinical opinion.^40^ If patients discontinued their first DMD, they could switch to another DMD or to best supportive care. After discontinuation of the second DMD, patients were assumed to be switching to best supportive care. The model takes a lifetime horizon: the effects of shared decision making on costs and QALYs are simulated until a patient dies or reaches the age of 100. A limited societal perspective was taken, in which health care costs and costs outside the health care sector (i.e., productivity losses related to MS, informal care, out-of-pocket or copayments for patients for equipment, aids and modifications, and community services, such as home help, transportation, or personal assistance) were included. A discount rate of 1.5% for effects and of 4.0% for costs was applied in accordance with the Dutch guideline for economic evaluations in health care.^36^ Table 1 presents key assumptions made in the model.

**Table 1:** Key Assumptions of the Health State Transition Model for MS Course by the Institute for Clinical and Economic Review and Key Assumptions for the Adapted Health State Transition Model Comparing Shared Decision Making with Usual Care

Assumptions of the health state transition model for MS course by Institute for Clinical and Economic Review^28,40^
• Mortality risk within each health state was the same for RRMS and SPMS.
• Patients progressing to SPMS continued treatment with DMD.
• Patients discontinue treatment if they progressed to a health state EDSS >6.
• The treatment effects of a DMD were equal for the first choice and the second choice.
• After discontinuation of second DMD, patients switch to best supportive care.
• No vial sharing was included.
Additional assumptions of the health state transition model comparing shared decision making with usual care
• Distribution of EDSS state of Dutch patients entering the model was equal to the original model.
• In usual care, decisions are mostly made in accordance with the health care professional’s judgment with little involvement of the patient’s preferences in the decision.
• Shared decision making affects initial treatment choice, DMD discontinuation, and DMD adherence.
• Effects of shared decision making on adherence and persistence remain stable over time.
• Nonadherent patients experience limited effects of the DMD.
• Discontinuation rates of the second DMD were equal to the discontinuation rate of the first DMD.
• Patients choosing best supportive care as initial treatment choice remained on best supportive care.

DMDs, disease-modifying drugs; EDSS, Expanded Disability Status Scale; MS, multiple sclerosis; RRMS, relapsing-remitting multiple sclerosis; SPMS, secondary progressive multiple sclerosis.

### Intervention Effects

**Figure 2:**
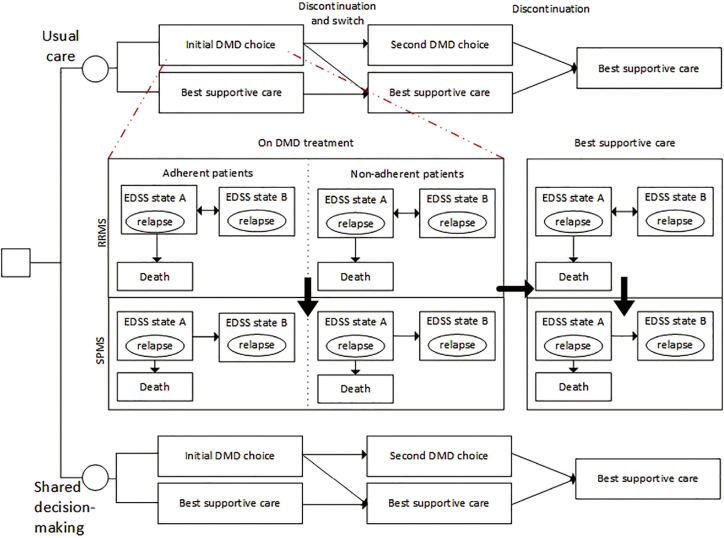
Health state transition model structure for the cost-effectiveness of shared decision making in multiple sclerosis. Patients diagnosed with relapsing-remitting multiple sclerosis (RRMS) receive either usual care or shared decision making to make a decision regarding treatment with disease-modifying drug. Three effects of shared decision making are modeled: 1) a change in initial treatment choice, 2) a decrease in discontinuation rate (persistence), and 3) an increase in the proportion of adherent patients. These effects are marked in the figure with a black circle and the corresponding number. If patients choose a drug treatment, they are either adherent (i.e., having more than 80% of days covered) or nonadherent. Nonadherent patients were assumed to have higher risks of experiencing (severe) relapses. For adherent and nonadherent patients, a simplified picture of the health state transition model for multiple sclerosis (MS) course is presented. The full model structure is provided in Figure 1. In short, patients enter the model in any of 10 RRMS-related health states based on the Expanded Disability Status Scale (EDSS). While the figure presents EDSS states A and B, these should be interpreted as the different EDSS levels (i.e., EDSS levels 0 through 10). During each 12-month cycle, the patients’ disability status could worsen, improve, or remain stable. Moreover, patients could progress to secondary progressive MS (SPMS). Patients could experience a relapse or die in any health state. If patients discontinue their initial treatment, they are assumed to switch to another active treatment or to best supportive care according to a predetermined probability. If patients discontinue their second treatment, they are assumed to be switching to best supportive care. DMD, disease-modifying drug.

Three effects of shared decision making were assumed and modeled (Figure 2 and further specified in the supplementary material): 1) shared decision making changes the DMD mix chosen, 2) shared decision making increases the proportion of optimally adherent patients, and 3) shared decision making increases persistence with the chosen treatment. Key assumptions for modeling shared decision making in comparison with usual care are presented in Table 1. Effect 1 was based on the fact that patients’ preferences are elicited and included in the treatment choice during a shared decision making process, and these preferences might differ from physicians’ preferences.^13^ The proportions of patients initiating a specific DMD treatment or opting for best supportive care were therefore assumed to differ between the groups, also following studies for other health decisions.^24,25,42,43^ The treatment initiation and treatment sequencing (i.e., the categorization of DMDs in first-line treatments for mild to moderate RRMS and in second-line treatments for highly active RRMS or RRMS not responding to first-line treatment) was informed by current clinical practice in the Netherlands, based on expert opinions and recommendations provided by the Dutch Healthcare Institute,^11^ in the absence of formal up-to-date clinical guidelines. Effects 2 and 3 were based on the theory by Stalmeier^26^ that patients develop a more pronounced attitude toward taking the medication continuously and accurately. Patients are expected to be better informed and consequently have more accurate expectations about the effects and treatment burden of the chosen treatment option.^26^ Consequently, a better match is made between the patient’s preferences and the treatment attributes. Patients on DMD treatment can be either optimally adherent or nonadherent over time. Nonadherent patients were assumed to not experience the full benefits of the DMDs and have a 42% higher risk of relapses overall^14^ and a 7.5% higher risk of severe relapses specifically.^44^ A weighted average of the outcomes was calculated.

#### Usual care profile

A profile for the usual care group was defined, and these parameters were kept constant for usual care in all further analyses. The usual care profile is presented in Table 2.

**Table 2:** Specification of the Profiles of Usual Care and Shared Decision Making per Treatment Based on the 3 Assumed Effects of Shared Decision Making^a^

	Effect 1, %		Effect 2, %		Effect 3, %	
	Treatment Initiation		Discontinuation Rate		Proportion Adherent	
	CAU	SDM	CAU	SDM	CAU	SDM
Best supportive care	25.0	20.0	NA	NA	100.0	100.0
Alemtuzumab	0.2	0.3	10.4	5.2	100.0	100.0
Dimethyl fumarate	33.6	41.3	21.4	10.7	58.9	63.9
Fingolimod	0.0	0.0	10.6	5.3	58.9	63.9
Glatiramer acetate 20 mg (generic)	0.2	0.1	26.7	13.4	58.9	63.9
Glatiramer acetate 20 mg (brand)	10.6	3.5	26.7	13.4	58.9	63.9
Glatiramer acetate 40 mg (brand)	0.2	0.1	21.5	10.8	58.9	63.9
Interferon β-1a	1.0	0.3	26.8	13.4	58.9	63.9
Interferon β-1a 22 mcg	0.5	0.2	27.1	13.6	58.9	63.9
Interferon β-1a 44 mcg	0.5	0.2	30.1	15.1	58.9	63.9
Interferon β-1b	1.0	0.3	25.9	13.0	58.9	63.9
Natalizumab	4.0	6.0	13.0	6.5	100.0	100.0
Ocrelizumab	0.8	1.2	13.1	6.6	100.0	100.0
Peginterferon β-1a	1.0	0.3	26.4	13.2	58.9	63.9
Teriflunomide 14 mg	21.4	26.3	20.8	10.4	58.9	63.9

CAU, care as usual; NA, not applicable; SDM, shared decision making.

a Different parameter values for the 3 effects of shared decision making were varied separately in sensitivity analyses. The values presented here were combined for the main base case scenario.

##### Treatment choice

Due to a lack of published data about the proportion of newly diagnosed patients choosing to start DMD treatment or best supportive care, rates were based on expert opinion, informed by data describing current use of each DMD by patients from the Drug Information System of the National Health Care Institute in the Netherlands.^45^

##### Discontinuation

Discontinuation rates of drug therapies reported in controlled phase II or III trials are most likely the optimal persistence rates among patients using the DMD but do not reflect real-world persistence as these are often lower.^15–17,46^ Therefore, discontinuation rates were based on real-world data, defined as more than 90 days’ interruption in treatment with the chosen DMD measured using pharmacy records.^15–17^ No data were available for ocrelizumab and alemtuzumab. Discontinuation rates were therefore determined based on the rate of natalizumab, proportionately according to differences found in trial discontinuation rates between ocrelizumab, alemtuzumab, and natalizumab. After discontinuation of the first DMD, patients could switch to another treatment or to best supportive care. Experts concluded that about 95% of patients who discontinued their first treatment would switch to another DMD treatment. From pivotal studies, it was estimated that 79%^47–57^ of patients discontinue due to side effects and 21%^48–53,56^ of patients discontinue due to a perceived lack of efficacy. Patients stopping first-line treatment due to side effects were assumed to switch to another first-line DMD. Patients stopping first-line treatment due to a perceived lack of efficacy were assumed to switch to a more effective (second-line) DMD (i.e., natalizumab, fingolimod, alemtuzumab, or ocrelizumab). If second-line DMD users discontinued treatment, they were assumed to switch to another second-line DMD (supplementary material).

##### Adherence

The proportion of adherent patients (patients with more than 80% of days covered) was measured using claim data and was set to 58.9% for self-injectable and oral DMDs in the usual care group.^14^ Since administration of alemtuzumab, ocrelizumab, and natalizumab takes place in the hospital, is less frequent, and is prescribed to patients with more active disease, 100% of patients taking these DMDs were assumed to have optimal adherence.

#### Shared decision making profile

The profile for shared decision making according to the 3 assumed effects was determined relative to the profile of usual care. The profile is specified in Table 2.

##### Effect 1: Treatment choice

Including the patient’s preferences in treatment decisions may change the choice.^24,25,42,43^ No evidence is yet available about how shared decision making could influence the treatment choice for MS. Based on stated preference studies, we constructed a possible profile regarding the initial treatment choice for the patients making a shared decision with their health care professional. Stated preference studies report that patients value the effects of the DMD most, more than safety, ease of use, and side effects.^12,58^ Moreover, oral medications have been reported to be preferred in comparison with injectable medications.^59–62^ Because first-line medications have similar effects and safety profiles, the profile specified that fewer patients start self-injectable first-line DMDs. Stated preference research further suggests that MS patients are less risk averse than neurologists^63^ and are willing to accept even higher risks than are currently associated with DMDs.^63,64^ Therefore, this profile specified that a slightly larger proportion of patients with relatively high disease activity at first treatment initiation would initiate a second-line treatment if the decision were shared between the patient and the health care professional. The proportion of patients starting each treatment option is specified in Table 2. A variation on the scenario was conducted as well in sensitivity analyses (i.e., assuming the change in treatment would be 50% smaller, resulting in a more conservative profile).

##### Effect 2: Discontinuation

Patients in the intervention group were assumed to be more persistent in using the chosen DMD because they are better informed about its efficacy and side effects, have been involved in the treatment decision, and there is a better match between the patient’s preferences and the chosen treatment.^26^ A relative decrease in the discontinuation rate of 50% was applied for the group receiving shared decision making compared with the group receiving usual care. This resulted in an averaged absolute decrease in discontinuation rate of 11.1% over the different DMDs, which was in line with a previous economic evaluation regarding a decision aid for shared decision making in osteoporosis assuming a 10% decrease in the discontinuation after shared decision making.^42^ The relative decrease in the discontinuation rate was varied to 25% and 75% in sensitivity analyses.

##### Effect 3: Adherence

In previous studies on patient decision aids for other shared health care decisions, improvement rates in the proportion of adherent patients varied between 0% and 50%.^42,43^ Because no data are available for MS, a conservative assumption was made regarding improvement for the proportion of adherent patients (i.e., a 5% improvement in comparison with usual care). This rate was increased in 1-way sensitivity analyses to 10% and up to 100% of patients being adherent.

#### Model inputs

##### Effect estimates

Estimates of effectiveness of DMDs in reducing relapses, slowing down disease progression, and transition probabilities between health states were in accordance with the model published by Zimmermann et al.^40^ based on a Bayesian network meta-analysis using 33 clinical trials (supplementary material). Age- and gender-specific background mortality rates were derived from Statistics Netherlands,^65^ which were adjusted for MS-specific mortality based on the EDSS score.^66^

##### Utilities

Utilities and disutilities associated with health states, relapses, and adverse events were in accordance with the original model (supplementary material).^28,40^

##### Costs

Costs were expressed in euros (1 euro = 1.16 US dollars: August 2018). All costs were, where necessary, inflated using the Consumer Price Index for August 2018.^67^

##### DMD costs

Costs of DMDs (supplementary material) were determined using the Pharmacy Purchase Price included in the database of the National Health Care Institute in the Netherlands (www.medicijnkosten.nl). A €14 dispensing fee for pharmacists was added for each first prescription and €7 for each subsequent prescription.^68^ Administration costs for alemtuzumab, natalizumab, and ocrelizumab were included since these DMDs are administered through intravenous infusion in the hospital. In the Netherlands, alemtuzumab requires hospitalization of patients during the treatment period, while natalizumab and ocrelizumab only require outpatient day treatment. Self-injectable or orally administrated DMDs do not incur additional administration costs. Costs related to monitoring recommended in the package inserts were included per DMD. Pretreatment monitoring costs were included in the first year of treatment. Any monitoring required after discontinuation, including an extra specialist visit, was added as well.^40^ Health care utilization unit costs were determined in accordance with the Dutch manual for economic evaluations based on reference prices established by the National Healthcare Institute^69^ or based on tariffs from the Dutch Healthcare Authority^70^ (see the supplementary material).

##### Adverse events costs

The costs of adverse events are covered by the Dutch “diagnosis-related-group” (DRG) system. However, these DRG rates are not publicly available. The costs of adverse events contribute only marginally to the total costs.^40^ Therefore, we first calculated the adverse events costs as proportion of the health care costs in the original model^40^ and applied the same percentage to calculate the adverse events costs as a proportion of the health care costs for the Dutch context in our model.

##### Health state costs

Health state costs for each EDSS health state were calculated by interpolating the EDSS costs reported by Uitdehaag et al.^71^ (supplementary material). From a societal perspective, health care costs (i.e., inpatient care, day admission, consultations, tests, and medications other than DMDs), community services, investments in equipment, aids or modifications, informal care, and productivity losses were included. For productivity losses, only short-term absence costs were included according to the friction cost method required by the Dutch economic evaluation guideline.^72^ A sensitivity analysis was conducted in which productivity losses also included the costs of long-term absence, disability, and early retirement, according to the human capital approach.^73^ Relapse costs were also derived from Uitdehaag et al.^71^

##### Costs of shared decision making

Estimating the costs of shared decision making is complicated due to the heterogeneous nature of how shared decision making may be implemented in clinical practice. It could involve training of health care providers, development (including [pilot] research into the effects), distribution and implementation of patient decision aids that could have many forms (paper-based or app-based, online, or computer-based patient decision aids), and additional health care provider (MS nurse and/or neurologist) time caused by additional consultations or increased consultation time.^23^ Although patient decision aids have been shown to extend consultation time with a median of 2.6 minutes,^23^ there are little data available about the costs of shared decision making, and no data are available for shared decision making in MS. Therefore, we assumed a mean cost per patient of €100, which would cover a scenario of an increase in consultation time of 50% (€51.77) and any costs for training of health care providers, development, and implementation of a patient decision aid up to €48.23 per patient. Shared decision making is assumed to be applied each time a treatment choice is made (i.e., patients discontinuing their first treatment will choose their second treatment again via shared decision making).

### Analyses

Analyses were conducted in Excel 2016 (Microsoft Corporation, Redmond, WA). Drug costs, adverse event costs, other costs within and outside the health care sector, total societal costs, QALYs, number of relapses, and life years were calculated separately for shared decision making and for usual care. This enabled the calculation of incremental cost effectiveness ratios (ICERs).

The 3 effects of shared decision making were first analyzed separately in comparison with usual care and were then assessed in a combined scenario: a 10% decrease in the discontinuation rate in comparison with usual care, a 5% increase in patients with optimal adherence in comparison with the usual care group, and initial treatment choices as specified in Table 2. Because of the heterogeneous nature of shared decision making in terms of consultation time, health care professionals involved, and supporting tools used, complicating the estimation of the costs of shared decision making, a threshold analysis was performed for each effect separately and for the 3 effects combined to determine the maximum costs of shared decision making at which the ICER would exceed the thresholds of €20,000/QALY and €50,000/QALY.^74^ For the combined profile of the 3 effects, we conducted further 1-way sensitivity analyses to evaluate the influence of several parameters on the ICER: drug costs (+20%; –20%), costs of implementing shared decision making (€0), mean age of RRMS onset (29; 45 years old), proportion male (18%; 40%), patient distribution over EDSS levels when entering the model (100% in EDDS level 1; 100% in EDSS level 4; –3.75% in EDSS levels 0–3 and +5% in EDSS levels 4–6), transition probabilities moving between health states (+10%; –10%), choice of initial DMD (equal distribution over first-line DMDs only, no best supportive care; equal distribution over first-line DMDs only, with 20% choosing best supportive care), choice for secondary DMD after first discontinuation (switch to second-line DMDs only with equal distribution; switch from first-line DMDs to first-line and second-line DMDs with equal distribution), discount rate (3%; 0% for both QALYs and costs), and the perspective taken (health care perspective, societal perspective using the human capital approach for calculating productivity losses). In addition, a probabilistic sensitivity analysis with 10,000 iterations was conducted for the combined effects, which was presented in a cost-effectiveness plane. A cost-effectiveness acceptability curve was constructed to present the probability that shared decision making would be cost-effective in comparison with usual care at a range of willingness-to-pay thresholds. Specifications for distributions of the effects of shared decision making are included in the supplementary material. Distributions of other parameters were selected in line with the model developed earlier.^40^

### Model Validation

Model transparency and internal validity were reviewed by 2 independent researchers (SP, XGLVP). Evaluation of internal validity consisted of internal testing and debugging using null input values—as recommended by the ISPOR-SMDM Modeling Good Research Practices Task Force^75^—for utilities and disutilities, probabilities of relapses, adverse events costs, discount rates, drug costs, all costs, and changes in the cohort size. In addition, cell-by-cell verification of input calculations and formulas was conducted to identify any errors. Last, external validity was evaluated by comparing life expectancy predicted by the model for the Dutch population with published estimates.

## Results

Modeling each effect of shared decision making separately showed that shared decision making resulted in higher QALYs and costs (Table 3). The associated ICERs ranged from €4384 to €315,555 per QALY gained.

**Table 3:** Cost-Effectiveness Results from Analyses of the 3 Assumed Potential Effects of Shared Decision Making

		Usual Care		Shared Decision Making		Incremental		
Modeled Effect of Shared Decision Making (Deviation from CAU^a^)		Total Costs	QALYs	Total Costs	QALYs	Δ Costs	Δ QALY	ICER
**Initial DMD choice**	**Table 2**	**€397,646**	**7.67**	**€402,551**	**7.88**	**€4904**	**0.21**	**€23,509**
	−50%^b^	€397,646	7.67	€400,181	7.78	€2535	0.10	€24,294
**Discontinuation rate**	**−50%**	**€397,646**	**7.67**	**€401,163**	**8.47**	**€3517**	**0.80**	**€4384**
	−25%	€397,646	7.67	€396,840	8.00	−€807	0.33	Dominant
	−75%	€397,646	7.67	€407,635	9.13	€9988	1.46	€6828
**Proportion of adherent patients**	**+5%**	**€397,646**	**7.67**	**€400,878**	**7.68**	**€3231**	**0.01**	**€315,555**
	+10%	€397,646	7.67	€403,972	7.69	€6325	0.02	€308,843
	100%	€397,646	7.67	€422,535	7.75	€24,889	0.08	€303,809
**Combined effects (base case)**	^a^	**€397,646**	**7.67**	**€417,655**	**8.79**	**€20,009**	**1.12**	**€17,875**

CAU, care as usual; DMD, disease-modifying drug; ICER, incremental cost-effectiveness ratio; QALYs, quality-adjusted life years.

a The 3 effects of shared decision making included in the model are initial treatment choice, discontinuation rate, and adherence. Bolded rows present the values of parameters included in the combined effect analysis. In usual care, 25% of people choose best supportive care, 5% choose a DMD indicated for highly active multiple sclerosis (natalizumab/alemtuzumab), and 70% choose one of the first-line DMDs (25% for a first-generation first-line DMD, 45% for a second generation first-line DMD). Discontinuation rates range between 10% and 31%, depending on the DMD. The proportion of patients with optimal adherence is 58.9%, except for natalizumab, alemtuzumab, and ocrelizumab, for which adherence is assumed to be 100%.

b Change in treatment choices was reduced with 50% compared to the change in treatment choice as specified in Table 2.

### Treatment Choice

A change in choice of the type of treatment as specified in Table 2 resulted in an increase in drug cost of €8288. Health state costs decreased by €3384, resulting in an overall cost increase. Overall QALYs increased (0.21 QALYs gained). A more conservative change in treatment choice resulted in similar but slightly smaller consequences.

### Discontinuation

Total costs increased if discontinuation rates dropped 50% due to a larger proportion of patients being persistent with their treatment choice, resulting in €15,275 in higher drug costs. On the other hand, patients also experienced more beneficial effects, resulting in 0.23 more life years, 0.29 less relapses, 0.80 more QALYs, and, consequently, €11,755 less in health state costs. Varying the relative reduction in discontinuation rate of DMDs from a 50% decrease to a 25% or 75% decrease affected the ICER slightly, resulting in an ICER of €4384 and €6828 per QALY gained, respectively, for a 50% and 75% reduction and for shared decision making being the dominant choice if the relative reduction would only be 25%.

### Adherence

A change in the proportion of adherent patients of 5% showed minimal beneficial effects (0.01 QALYs gained, 0.03 fewer relapses). This resulted in a major increase in the ICER to €315,555. Varying the adherence from 10% to 100% did not affect the ICER substantially.

### Combined Effects

Combination of all 3 effects resulted in an ICER of €17,875 per QALY gained. The increase in total costs of €20,009 was driven by increased drug costs of €36,678. The beneficial effects translated into a QALY gain of 1.12, reduction of relapses of 0.49, life years gained of 0.31, and a reduction in health state costs of €16,666. More detailed results for all effects separately and the combined effects are included in the supplementary material.

### Sensitivity Analyses

Sensitivity analyses of the separate effects showed that, if shared decision making was assumed to decrease the relative reduction in discontinuation rate by 25%, the total costs for the intervention group would be €807 less in comparison with the control group due to a decrease in health state costs of €4994 and an increase of drug costs of €4189, resulting in shared decision making being the dominant choice. In all scenarios, increased drug use was the main cost driver, while other costs within and outside the health care sector decreased (supplementary material).

**Figure 3:**
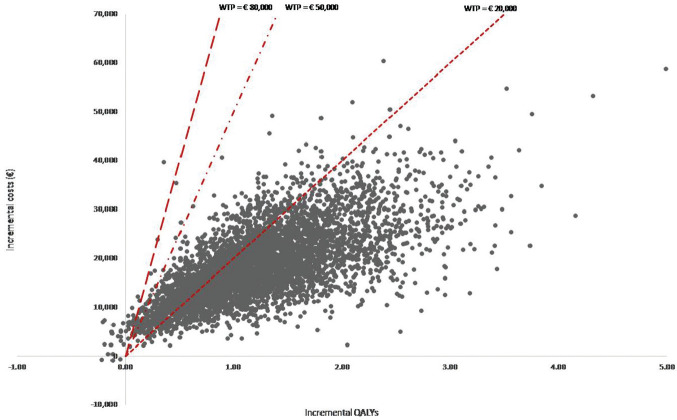
Incremental cost-effectiveness plane for the combined effects for initial disease-modifying drug choice, persistence, and adherence of shared decision making v. usual care. QALYs, quality-adjusted life years; WTP, willingness-to-pay threshold.

**Figure 4:**
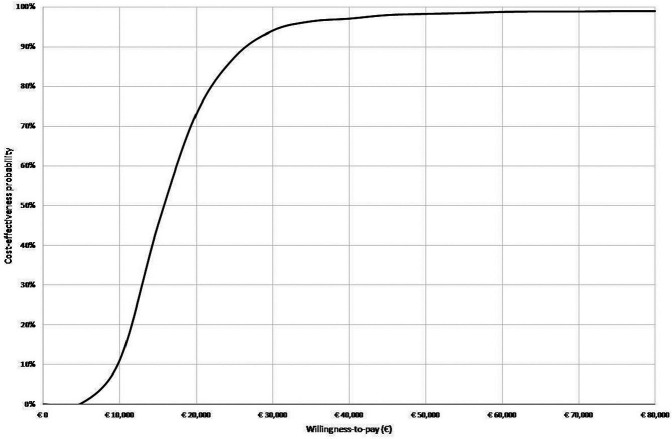
Cost-effectiveness acceptability curve for the combined effects for initial disease-modifying drug choice, persistence, and adherence of shared decision making v. usual care.

Results of 1-way sensitivity analyses of the combined effects as defined in Table 2 are presented in Table 4 (further detailed in the supplementary material). The ICER was highly sensitive to change in the relative risk of each DMD option for EDSS progression. Furthermore, the substantial influence of drug costs on the ICER was confirmed: a 20% increase and decrease in DMD costs resulted in ICERs of €24,147 and €11,604 per QALY gained, respectively. Moreover, the ICER increased to €25,568 and €29,191 if, respectively, a discount rate of 3% or a health care perspective was applied. A change in mean age of MS onset, the transition probabilities, and the choice for the initial and secondary treatment after the first discontinuation also affected costs and QALYs, resulting in ICERs ranging from €14,812 to €24,147. The threshold analyses suggest that, in the scenario of combined effects, shared decision making could maximally cost €23,639 to be cost-effective at a threshold of €50,000 per QALY (Table 5). Figure 3 presents the ICERs for the 10,000 iterations of the probabilistic analysis in a cost-effectiveness plane for the combined effects. The probability of shared decision making being cost-effective in comparison with usual care is 79.2% for a willingness-to-pay threshold of €20,000 per additional QALY, increasing to a probability above 98.5% and higher for thresholds of €50,000 and higher (Figure 4).

**Table 4:** Results of the 1-Way Sensitivity Analyses for the Combined Effects for Initial DMD Choice, Discontinuation, and Adherence^a^

	Usual care		Shared Decision Making		Shared Decision Making v. Usual Care		
Parameter	Total Cost	QALYs	Total Cost	QALYs	Δ Costs	Δ QALY	ICER
Base case (combined effects of shared decision making)	€397,646	7.67	€417,655	8.79	€20,009	1.12	€17,875
Drug costs +20%	€406,905	7.67	€433,934	8.79	€27,029	1.12	€24,147
Drug costs −20%	€388,387	7.67	€401,376	8.79	€12,989	1.12	€11,604
Relative risk for EDSS progression per DMD: lower range^b^	€384,371	8.58	€398,972	10.50	€14,601	1.91	€7,640
Relative risk for EDSS progression per DMD: upper range^b^	€413,913	6.53	€438,023	6.88	€24,110	0.34	€70,084
Costs of shared decision making: €0	€397,646	7.67	€417,502	8.79	€19,856	1.12	€17,739
Discount rate: 0%	€811,208	8.45	€839,286	9.98	€28,078	1.53	€18,307
Discount rate: 3%	€465,321	6.98	€486,766	7.82	€21,445	0.84	€25,568
Health care perspective	€203,427	7.67	€236,101	8.79	€32,675	1.12	€29,191
Human capital approach	€728,727	7.67	€744,564	8.79	€15,837	1.12	€14,149
Age at onset RRMS: 29	€447,395	7.17	€465,567	8.40	€18,173	1.23	€14,812
Age at onset RRMS: 45	€342,864	7.93	€364,618	8.91	€21,753	0.98	€22,285
Proportion male: 40%	€395,111	7.69	€415,203	8.81	€20,093	1.11	€18,046
Proportion male: 18%	€400,287	7.65	€420,208	8.78	€19,920	1.13	€17,700
EDSS level at start: 100% EDSS level 1	€370,333	9.29	€392,278	10.53	€21,946	1.24	€17,709
EDSS level at start: 100% EDSS level 4	€434,088	5.58	€451,517	6.54	€17,429	0.96	€18,132
EDSS level at start: equal distributions across health states	€393,989	6.59	€412,906	7.59	€18,917	1.00	€18,915
Choice initial DMD: only first-line DMD, equal proportions, no best supportive care	€397,646	7.67	€423,174	8.80	€25,527	1.12	€22,722
Choice initial DMD: only first-line DMD, 25% best supportive care	€406,905	7.67	€433,934	8.79	€27,029	1.12	€24,147
Choice second DMD: only second-line DMT with equal distributions	€399,151	8.17	€421,325	9.51	€22,174	1.34	€16,530
Choice second DMD: equal distributions across first line and second line	€396,515	7.83	€416,479	9.03	€19,964	1.20	€16,634
Transition probabilities: +10%	€403,414	7.21	€422,280	8.33	€18,866	1.13	€16,721
Transition probabilities: −10%	€391,241	8.20	€412,523	9.30	€21,283	1.11	€19,246

DMD, disease-modifying drug; EDSS, Expanded Disability Status Scale; ICER, incremental cost-effectiveness ratio; QALYs, quality-adjusted life years; RR, relative risk.

a As specified for usual care and shared decision making in Table 2.

b Range per DMD is specified in the supplementary material.

**Table 5:** Threshold Analyses for Maximum Costs of Shared Decision Making to Be Cost-Effective

		Threshold	
Effect	Size of Effect^a^	€20,000	€50,000
Initial treatment choice	Table 2	€−340.89	€3,428.17
Discontinuation rate	−50%	€8,440.43	€24,463.51
Proportion adherent	+5%	€−2,101.69	€−1,878.21
Combined effects		€1,656.86	€23,638.95

a Deviation from usual care.

### Validation results

Life expectancy predicted by the model varied from 33.7 to 36.1 years for the different DMD options and best supportive care. Considering a mean age of treatment start at 37 years, a life expectancy of 83.3 years for the general Dutch population,^76^ and a reduced life expectancy of 7 to 14 years for MS patients,^77^ the life expectancy estimated by the model (70.7 to 73.1 years) corresponds thus with published estimates.

## Discussion

Using a state transition model, this study suggests that shared decision making for DMDs for RRMS has the potential to be cost-effective, from a limited societal Dutch perspective, in comparison with usual care. Assuming that shared decision making would reduce discontinuation rates by 50%, increase the proportion of adherent patients by 5%, and lead to a slight increase in DMD initiation and the uptake of second-line and orally administered first-line DMDs, the ICER fell below accepted cost-effectiveness thresholds of €50,000 for diseases categorized as moderately burdensome in the Netherlands.^74^ The probability that the intervention would be cost-effective in comparison with usual care was 98.5% for a threshold of €50,000 per QALY. One-way sensitivity analyses showed that the ICER was robust for changes in costs of the intervention but more sensitive to variation in relative risk of progression for each DMD, drug costs, discount rates, or changes in perspectives. Nonetheless, all ICERs in the sensitivity analyses remained well below the commonly accepted threshold for cost-effectiveness of €50,000 per QALY gained,^74^ except when the relative risk of progression for each DMD would be considerably higher.

Threshold analyses showed that shared decision making will be cost-effective up to a maximum cost of €23,639 for a threshold of €50,000 per QALY, assuming the intervention would result in changes in initial treatment choice and a decrease in the discontinuation rate and the proportion of nonadherent patients. This maximum cost is very unlikely to be reached, as increase in consultation time would cost €104 per additional consultation, and training of health care professionals, development, and delivery of patient decision aids would in the worst-case scenario never add up to €23,639.

Two similar exploratory studies regarding the cost-effectiveness of shared decision making have been conducted, for obstructive sleep apnea and osteoporosis, and concluded that a patient decision aid could potentially be cost-effective.^42,43^ In contrast to our study, these studies found that the intervention’s cost of delivery had substantial influence on the cost-effectiveness of a patient decision aid, in addition to the influence of variations in the cost of the treatment.^43^ We found that the cost of shared decision making had little impact on the ICER. This could be explained by the considerable difference in treatment costs for obstructive sleep apnea and osteoporosis, which were only a fraction of the treatment costs for RRMS.

The actual effects of shared decision making for RRMS on treatment choice, persistence, adherence, and the cost of implementing the intervention are still largely unknown. Therefore, our study has an exploratory nature. Further trial- and registry-based evaluations would be needed to assess the real-life effects of shared decision making for MS. Modeling each potential effect of shared decision making separately allowed for assessment as to which of the 3 expected effects were most influential with regard to its cost-effectiveness and therefore informs further research and development of the intervention. Improvement in treatment adherence was found to have little effect on total QALYs. Various studies on enhancing adherence to medical intervention also failed to find a QALY and/or cost improvement with increased adherence rates—larger than the assumed adherence rate in our study—in trial-based economic evaluations.^78,79^ In contrast, the other 2 effects did affect the cost-effectiveness: a change in treatment initiation resulted in more incremental QALYs gained, and decreased discontinuation rates brought about the largest incremental QALY gain. If shared decision making would reduce the discontinuation by only 25%, increased drug costs are leveled out by reduced health state costs, causing shared decision making to be dominant over usual care, while with higher relative reductions in discontinuation rate, the drug cost increase exceeds the savings in health state costs. The importance of using shared decision making in decreasing discontinuation was also found for patient decision aids, which support shared decision making in osteoporosis.^42^ Our results suggest that the development of interventions to support shared decision making for RRMS should focus mainly on reducing the discontinuation of DMDs for the interventions to be cost-effective, since the type of treatment initiated is dependent on the patient’s preferences and needs and should not be directed by any intervention.

A strength of the current study is that a previously developed and validated model, used by regulatory bodies for policy decision making and pricing, was adapted to explore the cost-effectiveness of shared decision making in comparison with usual care. Moreover, a number of sensitivity analyses were conducted to establish the robustness of the results and conclusions. Inherent to the study design, however, is the simplification of real-life situations in a model. In addition to the limitations described by Zimmerman et al.^40^ concerning the quality and quantity of data available for the natural history of MS and DMD effectiveness, this model has some additional limitations. First, the model is based on the assumption that once patients choose best supportive care, as first, second, or third treatment course, they remain on best supportive care. In clinical practice, however, patients might choose to postpone treatment initially but decide after a while to start a DMD. This might underestimate the increase in drug costs as a consequence of shared decision making in comparison with usual care and thus, to some extent, the ICER. Further research is needed on whether shared decision making affects the probability of patients starting DMDs after initially opting for best supportive care. Second, nonadherence might increase over time, while in the model, nonadherence is assumed to be constant over time. This could overestimate the effect of shared decision making. Third, the mean age of RRMS onset, and thus the mean age of patients entering the model, might actually be lower. The estimate was based on cost-of-illness studies in the Netherlands, including patients who self-reported their disease course as RRMS, SPMS, and PPMS or don’t know. Patients may experience difficulty in identifying their disease course,^80^ and PPMS patients have on average a higher age of disease onset than RRMS and SPMS patients.^81^ Although a number of model-based economic evaluations included populations with similar mean ages at onset based on study samples from pivotal DMD studies, ranging from 36 to 38 years,^29,32,82–85^ some other studies included populations with mean ages of 29^40^ or 33^32^ years. Sensitivity analyses show that with lower ages of mean onset, shared decision making becomes more cost-effective, reducing the ICER with 17% (€3064) per QALY in comparison with the base case analysis. Fourth, cladribine has currently become available for patients with highly active RRMS or for patients who did not respond to a first-line DMD^11^ but was not included in the model as treatment option. Since cladribine received market authorization for highly active RRMS only in 2017 and was not included in the basic health insurance package in the Netherlands until March 2018, we expect that the share of patients currently choosing cladribine would be small and therefore have only a marginal impact on the results.

This exploratory study was specifically conducted for the Netherlands. Certain aspects of our model may limit the applicability of the results to contexts other than the Netherlands. For example, drug costs and recommendations for treatment sequencing (including the categorization in first-line and second-line therapy) differ between countries. In sensitivity analyses, we increased the drug cost of all DMDs by 20%, which showed that this parameter has a substantial effect on the ICER. In this scenario, the Dutch drug costs would still be 50% to 84% lower than the US drug costs.^40^ Shared decision making may, therefore, have more substantial impact on drug costs and the ICER in the United States. Moreover, assumed treatment sequencing was validated with Dutch clinicians, but clinical practice could differ in other countries^86^: for example, fingolimod is prescribed in the Netherlands only after another DMD has been ineffective but is approved as a first-line treatment option in the United States.

In conclusion, this study suggests that shared decision making for RRMS could potentially be a cost-effective intervention. Although shared decision making requires short-term investments in training staff, setting up structures for shared decision making and developing and/or acquiring patient decision aids, long-term savings should be considered in policy decisions, since threshold analyses show that shared decision making could be cost-effective even if upfront intervention cost are high. To our knowledge, this is the first study assessing the potential cost-effectiveness of shared decision making for RRMS. The current study could further inform the design of a trial-based study, which should be conducted to verify the assumptions made in the study and its results. This study provides insights for policy makers and clinicians regarding the potential value of implementing appropriate interventions to support shared decision making in treatment decisions for RRMS. Real-world current evidence on the cost-effectiveness of shared decision making is still limited^23^ and not available for RRMS. Real-world data on long-term treatment discontinuation rates after shared decision making should be collected.

## Supplemental Material

Supplementary_material_online_supp – Supplemental material for Exploring the Cost Effectiveness of Shared Decision Making for Choosing between Disease-Modifying Drugs for Relapsing-Remitting Multiple Sclerosis in the Netherlands: A State Transition Model Click here for additional data file. Supplemental material, Supplementary_material_online_supp for Exploring the Cost Effectiveness of Shared Decision Making for Choosing between Disease-Modifying Drugs for Relapsing-Remitting Multiple Sclerosis in the Netherlands: A State Transition Model by Ingrid E. H. Kremer, Mickael Hiligsmann, Josh Carlson, Marita Zimmermann, Peter J. Jongen, Silvia M. A. A. Evers, Svenja Petersohn, Xavier G. L. V. Pouwels and Nick Bansback in Medical Decision Making
