# Supplementary material for: Exploring the Cost Effectiveness of Shared Decision Making for Choosing between Disease-Modifying Drugs for Relapsing-Remitting Multiple Sclerosis in the Netherlands: A State Transition Model
Source: Med Decis Making. 2020 Nov 11;40(8):1003–19. doi: 10.1177/0272989X20961091 (PMC7672783; doi:10.1177/0272989X20961091)
Supplement: Supplementary_material_online_supp – Supplemental material for Exploring the Cost Effectiveness of Shared Decision Making for Choosing between Disease-Modifying Drugs for Relapsing-Remitting Multiple Sclerosis in the Netherlands: A State Transition Model [file Supplementary_material_online_supp.docx]

*Supplementary material*

**Exploring the cost-effectiveness of shared decision-making for choosing between disease-modifying drugs for multiple sclerosis in the Netherlands: a state transition model**

Kremer IEH, Hiligsmann M, Carlson J, Zimmermann M, Jongen PJ, Evers SMAA, Petersohn S, Pouwels XGLV, Bansback N.

**Content**

[1. Model structure 3](#_Toc21705631)

[1.1. Figure 1. Detailed model structure 3](#_Toc21705632)

[2. Parameters 4](#_Toc21705633)

[2.1. Table 1. Parameters derived from the model by the Institute for Clinical and Economic Review (1, 2) 4](#_Toc21705634)

[2.2. Table 2. Distribution of patients entering the model (Source: Zimmerman et al. 4](#_Toc21705635)

[2.3. Table 3. Relative Risk of EDSS Progression per treatment option 5](#_Toc21705636)

[2.4. Table 4. Healthcare utilization unit costs, range and standard error 6](#_Toc21705637)

[2.5. Table 5. Health state and relapse costs 7](#_Toc21705638)

[2.6. Table 6. Effects of shared decision-making (intervention) in comparison with usual care (control) 9](#_Toc21705639)

[2.7. Treatment initiation 11](#_Toc21705640)

[2.8. Table 7. Specification of treatment initiation in the intervention and control group 11](#_Toc21705641)

[2.9. Switching between treatments after first discontinuation 12](#_Toc21705642)

[3. Results 13](#_Toc21705643)

[3.1. Table 8. Results of scenario analyses and sensitivity analyses 13](#_Toc21705644)

[4. References 15](#_Toc21705645)

Model structure

## Figure 1. Detailed model structure

| 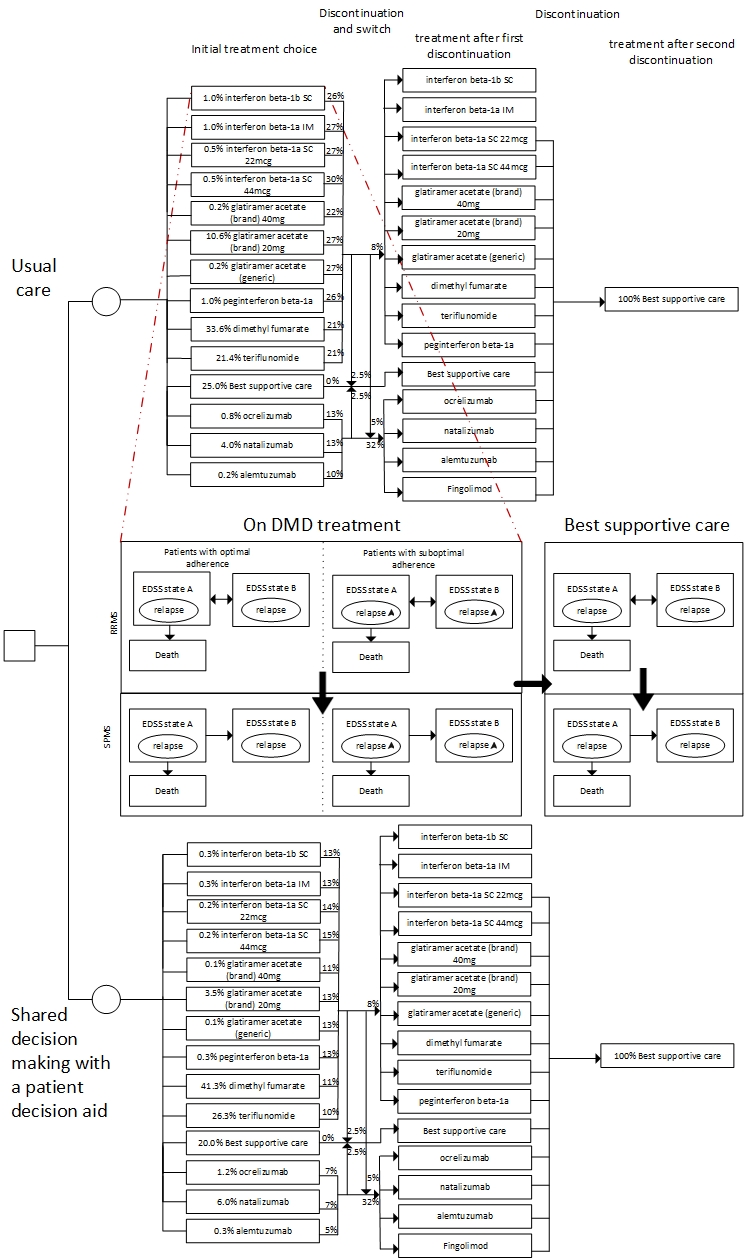 |
| --- |

# Parameters

## Table 1. Parameters derived from the model by the Institute for Clinical and Economic Review^1,2^

| Parameter |
| --- |
| Distribution of patients entering the model in each EDSS state |
| Probability of moving between EDSS states, RRMS and SPMS |
| Probability of conversion from RRMS tot SPMS |
| Annual Relapse rate, RRMS, SPMS |
| Probability of severe relapse (adherent patients) |
| Mortality multipliers of all-cause general population mortality |
| Annual disutility from adverse events |
| Healthcare resource utilization due to adverse events |
| Annual utility scores per health state, RRMS and SPMS |
| Disutility mild/moderate and severe relapses |
| Relative risk for EDSS progression |
| Rate ratio for relapse rate (adherent patients) |

- 1. Table 2. Distribution of patients entering the model (Source: Zimmerman et al.^2^)

| Health state^*^ | p | Range |
| --- | --- | --- |
| 1 | 0.22 | 0.17-0.26 |
| 2 | 0.28 | 0.23-0.34 |
| 3 | 0.24 | 0.19-0.29 |
| 4 | 0.15 | 0.12-0.18 |
| 5 | 0.06 | 0.05-0.07 |
| 6 | 0.00 | 0.00-0.00 |
| 7 | 0.00 | 0.00-0.00 |
| 8 | 0.00 | 0.00-0.00 |
| 9 | 0.00 | 0.00-0.00 |

^*^ Defined according to Expanded Disability Status Scale (EDSS)

## Table 3. Effectiveness of treatment options: risk of EDSS progression and risk of relapses in patients adherent and non-adherent to the treatment option

|  | Risk of EDSS progression^2^ | | Risk of relapses  Adherent patients^2^ | | Risk of relapses  Non-adherent patients^#^ | |
| --- | --- | --- | --- | --- | --- | --- |
|  |  |  |  |  |  |  |
| Treatment option | Relative risk | Range | Rate ratio | Range | Rate ratio | Range |
| Supportive Care | 1.00 |  | 1.00 |  | 1.00 |  |
| Alemtuzumab | 0.42 | 0.25-0.68 | 0.28 | 0.22-0.35 | NA | NA |
| Dimethyl fumarate | 0.62 | 0.46-0.84 | 0.53 | 0.43-0.63 | 0.73 | 0.63-0.83 |
| Fingolimod | 0.68 | 0.51-0.90 | 0.46 | 0.39-0.55 | 0.69 | 0.61-0.77 |
| Glatiramer acetate 20mg (generic®) | 0.74 | 0.58-0.94 | 0.63 | 0.55-0.71 | 0.79 | 0.71-0.87 |
| Glatiramer acetate 20mg (brand) | 0.74 | 0.58-0.94 | 0.63 | 0.55-0.71 | 0.79 | 0.71-0.87 |
| Glatiramer acetate 40mg (brand) | 1.17 | 0.69-1.92 | 0.67 | 0.52-0.86 | 0.81 | 0.64-0.98 |
| Interferon beta-1a IM | 0.79 | 0.63-1.00 | 0.83 | 0.74-0.94 | 0.90 | 0.80-1.00 |
| Interferon beta-1a 22mcg SC | 0.81 | 0.52-1.23 | 0.70 | 0.55-0.85 | 0.83 | 0.68-0.98 |
| Interferon beta-1a 44mcg SC | 0.73 | 0.52-0.99 | 0.64 | 0.54-0.73 | 0.79 | 0.70-0.89 |
| Interferon beta-1b | 0.66 | 0.46-0.89 | 0.65 | 0.55-0.77 | 0.80 | 0.69-0.91 |
| Natalizumab | 0.56 | 0.37-0.84 | 0.31 | 0.25-0.40 | NA | NA |
| Ocrelizumab | 0.47 | 0.28-0.76 | 0.35 | 0.27-0.44 | NA | NA |
| Peginterferon beta-1a | 0.63 | 0.37-1.02 | 0.63 | 0.47-0.86 | 0.79 | 0.59-0.98 |
| Teriflunomide | 0.72 | 0.52-0.97 | 0.67 | 0.56-0.79 | 0.81 | 0.69-0.92 |

^#^ Non-adherent patients were assumed to not experience the full benefits of the DMDs, and have a 42% higher risk of relapses overall.^3^ Since administration of alemtuzumab, ocrelizumab and natalizumab takes place in hospital, is less frequent and is prescribed to patients with more active disease, 100% of patients taking these DMDs were assumed to have optimal adherence.

EDSS, Expanded Disability Status Score; NA, not applicable.

## Table 4. Health state (dis)utilities and related to relapses (Source: Zimmerman et al.^2^)

|  | Base case | Range | Distribution for PSA |
| --- | --- | --- | --- |
| *Utilities* |  |  |  |
| *RRMS EDSS state* |  |  |  |
| 1 | 0.88 | 0.70-1.05 | Beta  Alpha=11.1, beta=1.6 |
| 2 | 0.83 | 0.67-1.00 | Beta  Alpha=15.1, beta=3.0 |
| 3 | 0.78 | 0.62-0.94 | Beta  Alpha=20.3, beta=5.7 |
| 4 | 0.69 | 0.56-0.83 | Beta  Alpha= 28.6, beta=12.6 |
| 5 | 0.63 | 0.50-0.75 | Beta  Alpha=35.4, beta=21.2 |
| 6 | 0.54 | 0.44-0.65 | Beta  Alpha=43.2, beta=36.2 |
| 7 | 0.46 | 0.36-0.55 | Beta  Alpha=51.8, beta=62.0 |
| 8 | 0.34 | 0.27-0.41 | Beta  Alpha=62.7, beta=119.7 |
| 9 | -0.17 | -0.014- -0.20 | Gamma  Alpha=96.04, beta=0.0 |
| Death | 0 | - |  |
| SPMSS EDSS State |  |  |  |
| 1 | 0.79 | 0.63-0.95 | Beta  Alpha=19.3, beta=5.1 |
| 2 | 0.74 | 0.59-0.88 | Beta  Alpha=24.6, beta=8.8 |
| 3 | 0.65 | 0.52-0.78 | Beta  Alpha=32.9, beta=17.6 |
| 4 | 0.58 | 0.47-0.70 | Beta  Alpha=39.6, beta=28.5 |
| 5 | 0.50 | 0.40-0.60 | Beta  Alpha=47.5, beta=47.4 |
| 6 | 0.41 | 0.33-0.49 | Beta  Alpha=56.1, beta=80.1 |
| 7 | 0.30 | 0.24-0.36 | Beta  Alpha=66.9, beta=156.2 |
| 8 | -0.04 | -0.03- -0.05 | Gamma  Alpha=96.04, beta=0.0 |
| 9 | -0.21 | -0.17- -0.26 | Gamma  Alpha=96.04, beta=0.0 |
| Death | 0 | - | - |
| *Disutilities* |  |  |  |
| Mild/moderate relapse | 0.09 | 0.07-0.11 | Beta  Alpha= 87.2, beta=871.1 |
| Severe relapse | 0.30 | 0.24-0.36 | Beta  Alpha= 66.7 beta=154.2 |

## Table 5. Healthcare utilization unit costs, range and standard error

| Parameter | Unit cost | Standard error |
| --- | --- | --- |
| *Drug costs*^4^ |  |  |
| Teriflunomide 14mg | €992.88 /28EA |  |
| Interferon beta-1a IM | €916.68 /4EA |  |
| Interferon beta-1b | €766.92 /14EA |  |
| Glatiramere acetate 20mg (brand) | €856.80 /30EA |  |
| Glatiramere acetate 40mg (brand) | €794.76 /30EA |  |
| Fingolimod | €1,916.70 /30EA |  |
| Glatiramere acetate 20mg (generic) | €771.00 /30EA |  |
| Alemtuzumab | €7,420.00 /1.2ml |  |
| Peginterferon beta-1a | €1,126.34 /1ml |  |
| Interferon beta-1a 22mcg SC | €868.56 /0.5ml |  |
| Interferon beta-1a 44 mcg SC | €1,076.88 /0.5ml |  |
| Dimethyl fumarate | €1,295.40 /60EA |  |
| Natalizumab | €1,670.58 /15ml |  |
| Ocrelizumab | €5,871.20 /300mg vial |  |
| Utilization costs |  |  |
| *Administration Costs*^5^ |  |  |
| Inpatient stay | €413.08 | 42.15 |
| Outpatient day treatment | €288.63 | 29.45 |
| *Laboratory Costs*^6^ |  |  |
| Complete blood count | €14.57 | 1.49 |
| Serum Creatinine | €3.52 | 0.36 |
| Urine analysis | €4.33 | 0.44 |
| Thyroid | €14.30 | 1.46 |
| Liver | €21.40 | 2.18 |
| MRI | €240.54 | 24.54 |
| ECG | €47.54 | 4.85 |
| ALT | €3.76 | 0.38 |
| Metabolic panel | €61.47 | 6.27 |
| *Visit Costs*^5^ |  |  |
| GP visit | €34.51 | 3.52 |
| Specialist visit | €103.53 | 10.56 |
| *Adverse event costs^#^*^2^ |  |  |
| PML | €26,008.09 | 2,653.89 |
| Hospital stay for disorders of the biliary without complications | €4,966.30 | 506.77 |
| Inpatient stay for depression | €4,308.94 | 439.69 |
| Hospital stay for influenza/pneumonia | €6,309.02 | 643.78 |
| Serious infection | €12,398.49 | 1,265.15 |
| Cranial nerve disorder | €8,685.00 | 886.22 |
| Patient decision aid costs^7^ | €100.00 | 10.20 |

* Normal distribution was applied for costs.

^#^ Estimation of adverse event costs is explained in the supplementary material.

GP, general practitioner; PML, Progressive multifocal leukoencephalopathy

Table 6. Health state and relapse costs

| Parameter | Unit cost | Range | Standard error |
| --- | --- | --- | --- |
| *EDSS state costs: healthcare costs* only^8^ | |  |  |
| 0 | €2,496 | 1997 -2996 | 255 |
| 1 | €3,539 | 2831 -4247 | 361 |
| 2 | €4,582 | 3665 -5498 | 468 |
| 3 | €5,624 | 4499 -6749 | 574 |
| 4 | €6,667 | 5333 -8000 | 680 |
| 5 | €7,709 | 6167 -9251 | 787 |
| 6 | €8,541 | 6833 -10250 | 872 |
| 7 | €9,303 | 7443 -11164 | 949 |
| 8 | €10,065 | 8052 -12079 | 1,027 |
| 9 | €10,828 | 8662 -12993 | 1,105 |
| *EDSS state costs: productivity costs (FCM), informal care, investments and services*^8^ | | | |
| 0 | €105 | 84 -125 | 11 |
| 1 | €1,118 | 894 -1342 | 114 |
| 2 | €2,131 | 1705 -2558 | 217 |
| 3 | €3,145 | 2516 -3774 | 321 |
| 4 | €4,158 | 3327 -4990 | 424 |
| 5 | €5,172 | 4137 -6206 | 528 |
| 6 | €9,398 | 7518 -11278 | 959 |
| 7 | €14,695 | 11756 -17634 | 1,500 |
| 8 | €19,993 | 15994 -23991 | 2,040 |
| 9 | €25,290 | 20232 -30348 | 2,581 |
| *EDSS state costs: productivity costs (HCA), informal care, investments and services*^8^ | | | |
| 0 | €11,322 | 9058-13586 | 1,155 |
| 1 | €13,373 | 10698-16047 | 1,365 |
| 2 | €15,423 | 12339-18508 | 1,574 |
| 3 | €17,474 | 13979-20969 | 1,783 |
| 4 | €19,525 | 15620-23430 | 1,992 |
| 5 | €21,576 | 17261-25891 | 2,202 |
| 6 | €27,122 | 21698-32546 | 2,768 |
| 7 | €33,833 | 27067-40600 | 3,452 |
| 8 | €40,545 | 32436-48654 | 4,137 |
| 9 | €47,256 | 37805-56707 | 4,822 |
| *Relapse costs: healthcare costs*^9^ |  |  |  |
| Mild/moderate | €2,042 | 1634-2451 | 208 |
| Severe | €2,042 | 1634-2451 | 208 |
| *Relapse costs: productivity costs^*^, informal care, investments and services*^9^ | | | |
| Mild/moderate | €1,052 | 842 -1263 | 107 |
| Severe | €1,052 | 842 -1263 | 107 |

Health state costs were calculated using interpolation of data from Uitdehaag et al.^8^ A separate formula was used to calculate health states costs of Expanded Disability Status Scores (EDSS) 0 to 5 (direct costs=1,002.7EDSS_x_ + 2,403; indirect costs=1,972.8EDSS_x_ + 10,891.8) and for EDSS 6 to 9 (direct costs=733.1EDSS_x_ + 3,818.3; indirect costs=6,456.4EDSS_x_ + 12,646.9) because of the categorization of costs in Uitdehaag et al.^8^

For probabilistic sensitivity analyses, a normal distribution was applied according to the previously published model^1,2^.

FCM, Friction Cost Method; HCA, Human Capital Approach

^*^ Early retirement, invalidity and DMD costs excluded

## Table 7. Effects of shared decision-making (intervention) in comparison with usual care (control)

| Parameter | Usual care | | | | | Shared decision-making | | | | |
| --- | --- | --- | --- | --- | --- | --- | --- | --- | --- | --- |
|  | Default value | Range | Standard error | alpha | beta | Default value | Range | Standard error | alpha | beta |
| *Treatment discontinuation rates* |  | | |  |  |  | | |  |  |
| Alemtuzumab (Lemtrada®) | 10.4%^10^ | 0.08 -0.12 | 0.01 | 85.95 | 740.47 | 0.4%^#^ |  |  |  |  |
| Dimethyl fumarate (Tecfidera®) | 21.4%^11^ | 0.17 -0.26 | 0.02 | 75.24 | 275.81 | 11.4%^#^ |  |  |  |  |
| Fingolimod (Gilenya®) | 10.6%^11^ | 0.09 -0.13 | 0.01 | 85.71 | 719.25 | 0.6%^#^ |  |  |  |  |
| Glatiramer acetate 20mg (Glatopa®) | 26.7%^12^ | 0.21 -0.32 | 0.03 | 70.13 | 192.53 | 16.7%^#^ |  |  |  |  |
| Glatiramer acetate 20mg (Copaxone®) | 26.7%^12^ | 0.21 -0.32 | 0.03 | 70.13 | 192.53 | 16.7%^#^ |  |  |  |  |
| Glatiramer acetate 40mg (Copaxone®) | 21.5%^12^ | 0.17 -0.26 | 0.02 | 75.18 | 274.48 | 11.5%^#^ |  |  |  |  |
| Interferon beta-1a (Avonex®) | 26.8%^12^ | 0.21 -0.32 | 0.03 | 70.03 | 191.28 | 16.8%^#^ |  |  |  |  |
| Interferon beta-1a 22mcg (Rebif®) | 27.1%^12^ | 0.22 -0.33 | 0.03 | 69.74 | 187.61 | 17.1%^#^ |  |  |  |  |
| Interferon beta-1a 44mcg (Rebif®) | 30.1%^12^ | 0.24 -0.36 | 0.03 | 66.83 | 155.20 | 20.1%^#^ |  |  |  |  |
| Interferon beta-1b (Betaseron®) | 25.9%^12^ | 0.21 -0.31 | 0.03 | 70.91 | 202.86 | 15.9%^#^ |  |  |  |  |
| Natalizumab (Tysabri®) | 13.0%^10^ | 0.10 -0.16 | 0.01 | 83.42 | 558.30 | 3.0%^#^ |  |  |  |  |
| Ocrelizumab (Ocrevus®) | 13.1%^10^ | 0.10 -0.16 | 0.01 | 83.33 | 552.76 | 3.1%^#^ |  |  |  |  |
| Peginterferon beta-1a (Plegridy®) | 26.4%^12^ | 0.21 -0.32 | 0.03 | 70.42 | 196.33 | 16.4%^#^ |  |  |  |  |
| Teriflunomide 14mg (Aubagio®) | 20.8%^11^ | 0.17 -0.25 | 0.02 | 75.82 | 288.13 | 10.8%^#^ |  |  |  |  |
| Relative reduction discontinuation rate |  |  |  |  |  | -50.0% | 0.25 -0.75 | 0.13 | 7.18 | 7.18 |
| *Proportion with suboptimal adherence* | 41.1%^3^ | 0.33-0.49 | 0.04 | 56.16 | 80.48 | -5%^#^ | 0.03 -0.08 | 0.01 | 14.55 | 276.41 |
| *Initial treatment choice* |  |  |  |  |  |  |  |  |  |  |
| Best supportive care | 25.0%^$^ | 0.20 -0.30 | 0.03 | 71.78 | 215.34 | 20.0%^#^ | 0.10-0.30 | 0.02 | 12.09 | 48.37 |
| Alemtuzumab (Lemtrada®) | 0.2%^$^ | 0.00 -0.00 | 0.00 | 95.85 | 47827.11 | 0.3%^#^ | 0.00-0.00 | 0.00 | 15.32 | 5090.45 |
| Dimethyl fumarate (Tecfidera®) | 33.6%^$^ | 0.27 -0.40 | 0.03 | 63.43 | 125.36 | 41.3%^#^ | 0.21-0.62 | 0.04 | 8.62 | 12.27 |
| Fingolimod (Gilenya®) | 0.0%^$^ | 0.00 -0.00 | 0.00 | 0.00 | 0.00 | 0.0%^#^ | 0.00-0.00 | 0.00 | 0.00 | 0.00 |
| Glatiramer acetate 20mg (Glatopa®) | 0.2%^$^ | 0.00 -0.00 | 0.00 | 95.85 | 47827.11 | 0.1%^#^ | 0.00-0.00 | 0.00 | 15.36 | 25076.77 |
| Glatiramer acetate 20mg (Copaxone®) | 10.6%^$^ | 0.08 -0.13 | 0.01 | 85.75 | 723.24 | 3.5%^#^ | 0.02-0.05 | 0.00 | 14.79 | 403.56 |
| Glatiramer acetate 40mg (Copaxone®) | 0.2%^$^ | 0.00 -0.00 | 0.00 | 95.85 | 47827.11 | 0.1%^#^ | 0.00-0.00 | 0.00 | 15.35 | 21702.93 |
| Interferon beta-1a (Avonex®) | 1.0%^$^ | 0.01 -0.01 | 0.00 | 95.07 | 9411.89 | 0.3%^#^ | 0.00-0.00 | 0.00 | 15.31 | 4578.70 |
| Interferon beta-1a 22mcg (Rebif®) | 0.5%^$^ | 0.00 -0.01 | 0.00 | 95.55 | 19015.41 | 0.2%^#^ | 0.00-0.00 | 0.00 | 15.34 | 9186.29 |
| Interferon beta-1a 44mcg (Rebif®) | 0.5%^$^ | 0.00 -0.01 | 0.00 | 95.55 | 19015.41 | 0.2%^#^ | 0.00-0.00 | 0.00 | 15.34 | 9186.29 |
| Interferon beta-1b (Betaseron®) | 1.0%^$^ | 0.01 -0.01 | 0.00 | 95.07 | 9411.89 | 0.3%^#^ | 0.00-0.00 | 0.00 | 15.31 | 4578.70 |
| Natalizumab (Tysabri®) | 4.0%^$^ | 0.03 -0.05 | 0.00 | 92.16 | 2211.80 | 6.0%^#^ | 0.03-0.09 | 0.01 | 14.38 | 225.36 |
| Ocrelizumab (Ocrevus®) | 0.8%^$^ | 0.03 -0.05 | 0.00 | 95.26 | 11812.70 | 1.2%^#^ | 0.01-0.02 | 0.00 | 15.17 | 1249.00 |
| Peginterferon beta-1a (Plegridy®) | 1.0%^$^ | 0.03 -0.05 | 0.00 | 95.07 | 9411.89 | 0.3%^#^ | 0.00-0.00 | 0.00 | 15.31 | 4578.70 |
| Teriflunomide 14mg (Aubagio®) | 21.4%^$^ | 0.09 -0.14 | 0.01 | 75.27 | 276.47 | 26.3%^#^ | 0.13-0.39 | 0.03 | 11.07 | 31.10 |

^#^ Based on assumptions

^$^ Based on expert opinion

## Treatment initiation

The proportions of patients initiating a specific DMD treatment or opting for best supportive care were assumed to differ among the groups, following studies for other health decisions.^7,13-15^

The mix of treatment initiations was informed by current clinical practice in the Netherlands based on expert opinions and recommendations provided by the Dutch Healthcare Institute,^16^ in the absence of formal up-to-date clinical guidelines. A patient’s eligibility for certain types of DMDs is determined by the degree of disease activity and whether previous DMD treatment has been unsuccessful in reducing relapses.^16^ Interferon beta-1a IM, interferon beta-1a 22 mcg and 44 mcg SC, interferon beta-1b SC, peginterferon beta-1a, glatiramer acetate (generic 20mg and brand 40 mg and 20 mg), teriflunomide and dimethyl fumarate are generally considered first-line treatments in the Netherlands. Natalizumab, alemtuzumab, fingolimod, ocrelizumab and cladribine are considered second-line treatments for patients with highly active MS because of their higher efficacy, but also less favourable safety profiles.^16^ Patients choosing for DMD treatment were assumed to either start first-line DMDs or, in the case of highly active MS at onset, directly start a DMD usually defined as a second-line treatment, with the exception of fingolimod. In the Netherlands, fingolimod can be prescribed only if treatment with another (first-line) DMD has been ineffective. Table 1 presents the proportion of patients initiating each treatment for usual care and different profiles of shared decision-making.

## Table 8. Specification of treatment initiation in the intervention and control group

|  | Treatment initiation | |  |
| --- | --- | --- | --- |
|  | CAU | SDM^#^ | SDM^$^ |
| Best supportive care | 25.0% | 20.0% | 22.5% |
| Alemtuzumab | 0.2% | 0.3% | 0.3% |
| Dimethyl fumarate | 33.6% | 41.3% | 37.4% |
| Fingolimod | 0.0% | 0.0% | 0.0% |
| Glatiramer acetate 20mg (generic) | 0.2% | 0.1% | 0.1% |
| Glatiramer acetate 20mg (brand) | 10.6% | 3.5% | 7.1% |
| Glatiramer acetate 40mg (brand) | 0.2% | 0.1% | 0.1% |
| Interferon beta-1a | 1.0% | 0.3% | 0.7% |
| Interferon beta-1a 22mcg | 0.5% | 0.2% | 0.3% |
| Interferon beta-1a 44mcg | 0.5% | 0.2% | 0.3% |
| Interferon beta-1b | 1.0% | 0.3% | 0.7% |
| Natalizumab | 4.0% | 6.0% | 5.0% |
| Ocrelizumab | 0.8% | 1.2% | 1.0% |
| Peginterferon beta-1a | 1.0% | 0.3% | 0.7% |
| Teriflunomide 14mg | 21.4% | 26.3% | 23.8% |

^#^ Profile in the combined scenario.

^$^ Values in the one-way sensitivity analyses

## Switching between treatments after first discontinuation

After discontinuation of the first DMD, patients could switch to another treatment or to best supportive care. Experts concluded that about 95% of patients who discontinued their first treatment would switch to another DMD treatment. From pivotal studies, it was estimated that 79%^17-27^ of patients discontinue due to side effects and 21%^18-23,26^ of patients discontinue due to a perceived lack of efficacy. The 5% of patients switching to best supportive care were assumed to be equally distributed between the patients discontinuing due to side effects and those discontinuing due to lack of efficacy. Patients stopping treatment due to side effects were assumed to switch to another first-line DMD, resulting in an 8% chance of switching from a first-line DMD to each of the other first-line DMDs (i.e. 79% of those discontinuing due to side effects switch to one of the other nine first-line DMDs – and 5%/2 switch to best supportive care). Patients stopping treatment due to a perceived lack of efficacy were assumed to switch to a second-line DMD, i.e. natalizumab, fingolimod, alemtuzumab or ocrelizumab, resulting in a 5% chance of switching from a first-line DMD to each second-line DMD (i.e. 21% of those discontinuing due to side effects switch to one of the four second-line DMDs, and 5%/2 switch to best supportive care). If second-line DMD-users discontinued treatment, they were assumed to switch to another second-line DMD (supplementary material).

# Results

## Table 9. Results of scenario analyses and sensitivity analyses

|  |  | Usual care | | | | | | | Shared decision making | | | | | | |  |  |  |
| --- | --- | --- | --- | --- | --- | --- | --- | --- | --- | --- | --- | --- | --- | --- | --- | --- | --- | --- |
|  | | total costs | Drug Costs | Direct and indirect cost | Adverse Event Costs | total QALYs | Relapses | Life-Years | total costs | Drug Costs | Direct and indirect cost | Adverse Event Costs | total QALYs | Relapses | Life-Years | ∆ total costs | ∆ QALY | ICER |
| *Effect of SDM vs. CAU ^*^* | |  |  |  |  |  |  |  |  |  |  |  |  |  |  |  |  |  |
| DMD choice | ^#^ | €397,646 | €47,986 | €349.650 | €10 | 7.67 | 15.28 | 26,59 | €402,551 | €56,274 | €346.266 | 11 | 7.88 | 15.19 | 26,64 | €4,904 | 0.21 | €23,509 |
|  | ^&^ | €397,646 | €47,986 | €349.650 | €10 | 7.67 | 15.28 | 26,59 | €400,181 | €52,130 | €348.040 | 10 | 7.78 | 15.23 | 26,62 | €2,535 | 0.10 | €24,294 |
| Discontinuation rate | -10% | €397,646 | €47,986 | €349.650 | €10 | 7.67 | 15.28 | 26,59 | €401,163 | €63,261 | €337.896 | 6 | 8.47 | 14.99 | 26,82 | €3,517 | 0.80 | €4,384 |
|  | -5% | €397,646 | €47,986 | €349.650 | €10 | 7.67 | 15.28 | 26,59 | €396,840 | €52,176 | €344.656 | 8 | 8.00 | 15.17 | 26,68 | -€807 | 0.33 | DOMINANT |
|  | -20% | €397,646 | €47,986 | €349.650 | €10 | 7.67 | 15.28 | 26,59 | €407,635 | €78,373 | €329.257 | 4 | 9.13 | 14.70 | 27,03 | €9,988 | 1.46 | €6,828 |
| Proportion adherent | +5% | €397,646 | €47,986 | €349.650 | €10 | 7.67 | 15.28 | 26,59 | €400,878 | €51,152 | €349.715 | 10 | 7.68 | 15.25 | 26,59 | €3,231 | 0.01 | €315,555 |
|  | +10% | €397,646 | €47,986 | €349.650 | €10 | 7.67 | 15.28 | 26,59 | €403,972 | €54,318 | €349.643 | 10 | 7.69 | 15.23 | 26,59 | €6,325 | 0.02 | €308,843 |
|  | 100% | €397,646 | €47,986 | €349.650 | €10 | 7.67 | 15.28 | 26,59 | €422,535 | €73,314 | €349.211 | 10 | 7.75 | 15.06 | 26,59 | €24,889 | 0.08 | €303,809 |
| Combined effects |  | €397,646 | €47,986 | €349.650 | €10 | 7.67 | 15.28 | 26,59 | €417,655 | €84,664 | €332.984 | 7 | 8.79 | 14.79 | 26,90 | €20,009 | 1.12 | €17,875 |
| *Sensitivity analyses: combined effects* | |  | | | | | | |  | | | | | | |  | | |
| Drug costs +20% | | €406,905 | €57,245 | €349,650 | €10 | 7.67 | 15.28 | 26.59 | €433,934 | €100,943 | €332,984 | 7 | 8.79 | 14.79 | 26.90 | €27,029 | 1.12 | €24,147 |
| Drug costs -20% | | €388,387 | €38,727 | €349,650 | €10 | 7.67 | 15.28 | 26.59 | €401,376 | €68,385 | €332,984 | 7 | 8.79 | 14.79 | 26.90 | €12,989 | 1.12 | €11,604 |
| Relative risk progression rate EDSS states: improvement | | €384,371 | €49,886 | €334,475 | €10 | 8.58 | 15.84 | 26.85 | €398,972 | €91,725 | €307,240 | 6 | 10.50 | 15.70 | 27.42 | €14,601 | 1.91 | €7,640 |
| Relative risk progression rate EDSS states: worsening | | €413,913 | €44,895 | €369,007 | €11 | 6.53 | 14.62 | 26.27 | €438,023 | €74,661 | €363,355 | 7 | 6.88 | 13.89 | 26.34 | €24,110 | 0.34 | €70,084 |
| Costs pDA: €0 | | €397,646 | €47,986 | €349,650 | €10 | 7.67 | 15.28 | 26.59 | €417,502 | €84,664 | €332,832 | 7 | 8.79 | 14.79 | 26.90 | €19,856 | 1.12 | €17,739 |
| Discount rate: 3% | | €465,321 | €50,231 | €415,077 | €12 | 6.98 | 15.28 | 21.29 | €486,766 | €90,935 | €395,823 | 8 | 7.82 | 14.79 | 21.48 | €21,445 | 0.84 | €25,568 |
| Discount rate: 0% | | €811,208 | €58,601 | €752,584 | €23 | 8.45 | 15.28 | 34.28 | €839,286 | €116,807 | €722,464 | 15 | 9.98 | 14.79 | 34.80 | €28,078 | 1.53 | €18,307 |
| Healthcare perspective | | €203,427 | €47,986 | €155,436 | €5 | 7.67 | 15.28 | 26.59 | €236,101 | €84,664 | €151,434 | 3 | 8.79 | 14.79 | 26.90 | €32,675 | 1.12 | €29,191 |
| Human capital approach | | €728,727 | €47,986 | €680,721 | €20 | 7.67 | 15.28 | 26.59 | €744,564 | €84,664 | €659,887 | 13 | 8.79 | 14.79 | 26.90 | €15,837 | 1.12 | €14,149 |
| age at onset RRMS: 29 | | € 447,395 | € 48,141 | € 399,242 | € 11.67 | 7.17 | 17.51 | 30.18 | € 465,567 | € 85,338 | € 380,221 | € 8 | 8.40 | 17.02 | 30.45 | € 18,173 | 1.23 | € 14,812 |
| age at onset RRMS: 45 | | € 342,864 | € 47,627 | € 295,229 | € 8.63 | 7.93 | 13.12 | 22.92 | € 364,618 | € 83,246 | € 281,366 | € 5.72 | 8.91 | 12.62 | 23.26 | € 21,753 | 1.23 | € 22,285 |
| Proportion male: 40% | | € 395,111 | € 47,975 | € 347,125 | € 10.14 | 7.69 | 15.17 | 26.41 | € 415,203 | € 84,620 | € 330,577 | € 6.73 | 8.81 | 14.68 | 26.73 | € 20,093 | 1.11 | € 18,046 |
| Proportion male: 18% | | € 400,287 | € 47,997 | € 352,280 | € 10.29 | 7.65 | 15.39 | 26.78 | € 420,208 | € 84,708 | € 335,493 | € 6.83 | 8.78 | 14.90 | 27.09 | € 19,920 | 1.13 | € 17,700 |
| EDSS level at start: 100% EDSS level 1 | | € 370,333 | € 50,147 | € 320,179 | € 7.03 | 9.29 | 16.26 | 27.07 | € 392,278 | € 90,364 | € 301,910 | € 3.43 | 10.53 | 15.69 | 27.41 | € 21,946 | 1.24 | € 17,709 |
| EDSS level at start: 100% EDSS level 4 | | € 434,088 | € 45,307 | € 388,773 | € 8.51 | 5.58 | 14.07 | 26.06 | € 451,517 | € 77,391 | € 374,122 | € 4.15 | 6.54 | 13.69 | 26.32 | € 17,429 | 0.96 | € 18,132 |
| EDSS level at start: equal distribution across health states | | € 393,989 | € 44,475 | € 349,499 | € 15.71 | 6.59 | 14.16 | 25.22 | € 412,906 | € 77,424 | € 335,469 | € 13.52 | 7.59 | 13.76 | 25.54 | € 18,917 | 1.00 | € 18,915 |
| Choice initial DMD: only first line DMD, equal proportions, no best supportive care | | € 397,646 | € 47,986 | € 349,650 | € 10.22 | 7.67 | 15.28 | 26.59 | € 423,174 | € 90,749 | € 332,417 | € 8.44 | 8.80 | 14.66 | 26.90 | € 25,527 | 1.12 | € 22,722 |
| Choice initial DMD: only first line DMD, 25% best supportive care | | € 406,905 | € 57,245 | € 349,650 | € 10.22 | 7.67 | 15.28 | 26.59 | € 433,934 | € 100,943 | € 332,984 | € 6.78 | 8.79 | 14.79 | 26.90 | € 27,029 | 1.12 | € 24,147 |
| Choice second DMD: only second line DMT with equal distributions | | € 399,151 | € 58,005 | € 341,144 | € 2.78 | 8.17 | 15.01 | 26.71 | € 421,325 | € 99,258 | € 322,064 | € 3.11 | 9.51 | 14.31 | 27.09 | € 22,174 | 1.34 | € 16,530 |
| Choice second DMD: equal distributions across first-line and second-line | | € 396,515 | € 49,556 | € 346,950 | € 9.47 | 7.83 | 15.23 | 26.63 | € 416,479 | € 87,118 | € 329,355 | € 6.40 | 9.03 | 14.70 | 26.97 | € 19,964 | 1.20 | € 16,634 |
| Transition probabilities: +10% | | € 403,414 | € 47,600 | € 355,804 | € 10.39 | 7.21 | 15.01 | 26.40 | € 422,280 | € 83,431 | € 338,843 | € 6.89 | 8.33 | 14.56 | 26.71 | € 18,866 | 1.13 | € 16,721 |
| Transition probabilities: -10% | | € 391,241 | € 48,383 | € 342,847 | € 10.03 | 8.20 | 15.59 | 26.81 | € 412,523 | € 85,973 | € 326,544 | € 6.65 | 9.30 | 15.06 | 27.12 | € 21,283 | 1.11 | € 19,246 |

^*^In usual care, 25% of people choose best supportive care, 5% choose a DMD indicated for highly active MS (natalizumab/alemtuzumab) and 70% choose one of the first-line DMDs (25% for a first-generation first-line DMD, 45% for a second generation first-line DMD). Discontinuation rates range between 10% and 31%, depending on the DMD. The proportion of patients with optimal adherence is 60%, except for natalizumab, alemtuzumab and ocrelizumab for which adherence is assumed to be 100%.

^#^ Specified in Table 4.

^&^ Specification of change in treatment initiation as specified in Table 4 decreased by 50%.

BC, base case; OSA, one-way sensitivity analysis.

# References

[1] Institute for Clinical and Economic Review. Disease-modifying therapies for relapsing-remitting and primary-progressive multiple sclerosis: effectiveness and value [Internet]. 2017 [cited 2018 December 14] Available from: https://icer-review.org/wp-conte nt/uploa ds/2016/08/CTAF_MS_FinalReport_030617.pdf

[2] Zimmermann M, Brouwer E, Tice JA, Seidner M, Loos AM, Liu S, et al. Disease-modifying therapies for relapsing-remitting and primary progressive multiple sclerosis: a cost-utility analysis. CNS drugs. 2018.

[3] Burks J, Marshall TS, Ye X. Adherence to disease-modifying therapies and its impact on relapse, health resource utilization, and costs among patients with multiple sclerosis. Clinicoecon Outcomes Res. 2017; 9:251-60.

[4] Zorginstituut Nederland. Medicijnkosten. [cited 2018 October 16] Available from: www.medicijnkosten.nl

[5] Hakkaart-van Roijen L, van der Linden N, Bouwmans C, Kanters T, Tan SS. Kostenhandleiding: Methodologie van kostenonderzoek en referentieprijzen voor economische evaluaties in de gezondheidszorg. Rotterdam, The Netherlands: Institute for Medical Technology Assessment, Erasmus Universiteit Rotterdam; 2015.

[6] Nederlandse Zorgautoriteit (NZa). DBC zorgproducten tariefapplicatie. . 2018 [cited 2018 October 16] Available from: https://zorgproducten.nza.nl/

[7] Penton H, Hiligsmann M, Harrison M, Reginster JY, Boonen A, Bansback N. Potential cost-effectiveness for using patient decision aids to guide osteoporosis treatment. Osteoporos Int. 2016; 27(9):2697-707.

[8] Uitdehaag B, Kobelt G, Berg J, Capsa D, Dalen J. New insights into the burden and costs of multiple sclerosis in Europe: results for the Netherlands. Mult Scler. 2017; 23(2_suppl):117-29.

[9] Kobelt G, Berg J, Lindgren P, Anten B, Ekman M, Jongen PJ, et al. Costs and quality of life in multiple sclerosis in The Netherlands. Eur J Health Econ. 2006; 7 Suppl 2:S55-64.

[10] Kramer J, Tenberge JG, Kleiter I, Gaissmaier W, Ruck T, Heesen C, et al. Is the risk of progressive multifocal leukoencephalopathy the real reason for natalizumab discontinuation in patients with multiple sclerosis? PLoS One. 2017; 12(4):e0174858.

[11] Setayeshgar S, Kingwell E, Zhu F, Zhang T, Carruthers R, Marrie RA, et al. Persistence and adherence to the new oral disease-modifying therapies for multiple sclerosis: a population-based study. Mult Scler Relat Disord. 2018; 27:364-9.

[12] Evans C, Marrie RA, Zhu F, Leung S, Lu X, Melesse DY, et al. Adherence and persistence to drug therapies for multiple sclerosis: a population-based study. Mult Scler Relat Disord. 2016; 8:78-85.

[13] Arterburn D, Wellman R, Westbrook E, Rutter C, Ross T, McCulloch D, et al. Introducing decision aids at Group Health was linked to sharply lower hip and knee surgery rates and costs. Health aff (Milwood). 2012; 31(9):2094-104.

[14] Trenaman L, Sadatsafavi M, Almeida F, Ayas N, Lynd L, Marra C, et al. Exploring the Potential Cost-Effectiveness of Patient Decision Aids for Use in Adults with Obstructive Sleep Apnea: A Case Study. Med Decis Making. 2015; 35(5):671-82.

[15] van Peperstraten A, Nelen W, Grol R, Zielhuis G, Adang E, Stalmeier P, et al. The effect of a multifaceted empowerment strategy on decision making about the number of embryos transferred in in vitro fertilisation: randomised controlled trial. BMJ. 2010; 341:c2501.

[16] Zorginstituut Nederland. Farmacotherapeutisch Kompas. [cited 2018 October 16] Available from: https://www.farmacotherapeutischkompas.nl/

[17] Calabresi PA, Kieseier BC, Arnold DL, Balcer LJ, Boyko A, Pelletier J, et al. Pegylated interferon beta-1a for relapsing-remitting multiple sclerosis (ADVANCE): a randomised, phase 3, double-blind study. Lancet Neurol. 2014; 13(7):657-65.

[18] Calabresi PA, Radue EW, Goodin D, Jeffery D, Rammohan KW, Reder AT, et al. Safety and efficacy of fingolimod in patients with relapsing-remitting multiple sclerosis (FREEDOMS II): a double-blind, randomised, placebo-controlled, phase 3 trial. Lancet Neurol. 2014; 13(6):545-56.

[19] Coles AJ, Twyman CL, Arnold DL, Cohen JA, Confavreux C, Fox EJ, et al. Alemtuzumab for patients with relapsing multiple sclerosis after disease-modifying therapy: a randomised controlled phase 3 trial. Lancet. 2012; 380(9856):1829-39.

[20] Gold R, Kappos L, Arnold DL, Bar-Or A, Giovannoni G, Selmaj K, et al. Placebo-controlled phase 3 study of oral BG-12 for relapsing multiple sclerosis. N Engl J Med. 2012; 367(12):1098-107.

[21] Hauser SL, Bar-Or A, Comi G, Giovannoni G, Hartung HP, Hemmer B, et al. Ocrelizumab versus interferon beta-1a in relapsing multiple sclerosis. N Engl J Med. 2017; 376(3):221-34.

[22] Khan O, Rieckmann P, Boyko A, Selmaj K, Zivadinov R. Three times weekly glatiramer acetate in relapsing-remitting multiple sclerosis. Ann Neurol. 2013; 73(6):705-13.

[23] Mikol DD, Barkhof F, Chang P, Coyle PK, Jeffery DR, Schwid SR, et al. Comparison of subcutaneous interferon beta-1a with glatiramer acetate in patients with relapsing multiple sclerosis (the REbif vs Glatiramer Acetate in Relapsing MS Disease [REGARD] study): a multicentre, randomised, parallel, open-label trial. Lancet Neurol. 2008; 7(10):903-14.

[24] O'Connor P, Filippi M, Arnason B, Comi G, Cook S, Goodin D, et al. 250 microg or 500 microg interferon beta-1b versus 20 mg glatiramer acetate in relapsing-remitting multiple sclerosis: a prospective, randomised, multicentre study. Lancet Neurol. 2009; 8(10):889-97.

[25] Polman CH, O'Connor PW, Havrdova E, Hutchinson M, Kappos L, Miller DH, et al. A randomized, placebo-controlled trial of natalizumab for relapsing multiple sclerosis. N Engl J Med. 2006; 354(9):899-910.

[26] Vermersch P, Czlonkowska A, Grimaldi LM, Confavreux C, Comi G, Kappos L, et al. Teriflunomide versus subcutaneous interferon beta-1a in patients with relapsing multiple sclerosis: a randomised, controlled phase 3 trial. Mult Scler. 2014; 20(6):705-16.

[27] Vollmer TL, Sorensen PS, Selmaj K, Zipp F, Havrdova E, Cohen JA, et al. A randomized placebo-controlled phase III trial of oral laquinimod for multiple sclerosis. J Neurol. 2014; 261(4):773-83.
